# Carcinome à cellules claires du col de l’utérus en Mauritanie: à propos d’un cas

**DOI:** 10.11604/pamj.2026.53.32.49183

**Published:** 2026-01-26

**Authors:** Ahmed El Heiba Mamina, Khadijetou Vilaly, Ekht Elbenina Zein, Mohamed Nouh Memah, El Hadj Menny, Ahmedou Tolba, Mouna Mohamed Yaya Elkherchy

**Affiliations:** 1 Faculté de Médecine, de Pharmacie et d'Odonto-Stomatologie, Service d'Anatomie et de Cytologie Pathologique, Centre National d'Oncologie, Nouakchott, Mauritanie,; 2 Faculté de Médecine, de Pharmacie et d'Odonto-Stomatologie, Centre Hospitalier Mère et Enfant, Nouakchott, Mauritanie,; 3 Faculté de Médecine, de Pharmacie et d'Odonto-Stomatologie, Centre National d'Oncologie, Nouakchott, Mauritanie,; 4 Centre Hospitalier National, Nouakchott, Mauritanie

**Keywords:** Carcinome à cellules claires, cancer du col de l’utérus, radiochimiothérapie, Mauritanie, cas clinique, Clear cell carcinoma, cervical cancer, chemoradiotherapy, Mauritania, case report

## Abstract

Le carcinome à cellules claires (CCC) du col de l'utérus est un cancer rare, surtout en l'absence d'exposition au diéthylstilbestrol (DES). Ce cas est unique car il concerne une patiente de 40 ans sans antécédents d'exposition au DES, illustrant les défis diagnostiques et thérapeutiques dans un contexte à ressources limitées, comme la Mauritanie. La patiente présentait des saignements vaginaux persistants, avec une masse détectée lors d'un examen gynécologique. Le diagnostic final de CCC a été confirmé par des examens histopathologiques et immunohistochimiques. Le traitement a consisté en une radiothérapie externe et une chimiothérapie, suivies d'une colpohystérectomie élargie, sans recours à la curiethérapie en raison de contraintes techniques. La patiente a bien répondu au traitement avec une absence de récidive clinique et radiologique. Ce cas met en évidence la faisabilité d'une prise en charge multidisciplinaire efficace des tumeurs rares du col de l'utérus dans des contextes à ressources limitées.

## Abstract (en)

Clear cell carcinoma (CCC) of the cervix is a rare cancer, particularly in the absence of exposure to diethylstilbestrol (DES). This case is unique because it involves a 40-year-old patient with no history of DES exposure, illustrating the diagnostic and therapeutic challenges in a resource-limited setting such as Mauritania. The patient presented with persistent vaginal bleeding, and a mass was detected during a gynaecological examination. The final diagnosis of CCC was confirmed by histopathological and immunohistochemical examination. Treatment consisted of external radiotherapy and chemotherapy, followed by an extended colpohysterectomy, without the use of brachytherapy because of technical constraints. The patient responded well to treatment, with no clinical or radiological evidence of recurrence. This case underscores the feasibility of effective multidisciplinary management of rare cervical tumors in resource-limited settings.

## Introduction

Le cancer du col de l'utérus est le cancer gynécologique le plus fréquent à l'échelle internationale [1]. En Mauritanie, il se classe au deuxième rang après le cancer du sein [2]. Les sous-types histologiques les plus courants sont le carcinome épidermoïde (82,8%) et l'adénocarcinome (9,6%) [3]. Le CCC est un type rare de cancer, représentant environ 4% des adénocarcinomes cervicaux. Historiquement, il est principalement associé à l'exposition intra-utérine au diéthylstilbestrol (DES), bien que des cas aient été signalés chez des femmes sans antécédents d'exposition au DES, y compris chez des enfants et des adultes. Ce type de carcinome peut également se développer dans d'autres sites du tractus génital féminin, tels que le vagin, l'endomètre et les ovaires [1].

Ce cas clinique concerne une patiente de 40 ans sans exposition connue au DES, illustrant la complexité du diagnostic et de la prise en charge thérapeutique dans le contexte mauritanien ainsi que la spécificité de cette maladie. L'objectif de ce rapport est de présenter les caractéristiques cliniques, les méthodes diagnostiques et les options thérapeutiques du carcinome à cellules claires du col de l'utérus, tout en contribuant à la littérature limitée sur le sujet, notamment en Mauritanie et plus largement en Afrique. Ce travail vise à fournir des informations utiles pour orienter les futurs protocoles de prise en charge.

## Patient et observation

**Présentation de la patiente:** la patiente, âgée de 40 ans, s'est présentée avec des saignements vaginaux irréguliers. Elle est atteinte d'hypothyroïdie, diagnostiquée deux ans auparavant, et est sous traitement substitutif. Elle n'a pas d'antécédents familiaux de cancer connus. Sa ménarche a eu lieu à l'âge de 14 ans, et elle avait des cycles menstruels réguliers jusqu'à l'apparition des symptômes actuels. Elle ne présente aucun antécédent d'exposition intra-utérine au DES. Les symptômes ont débuté après un épisode d'avortement spontané, associé à des pertes blanchâtres et des saignements persistants, motivant une consultation gynécologique.

**Chronologie:** en janvier 2024, la patiente a présenté un épisode d'avortement spontané suivi de saignements persistants et de pertes blanchâtres, conduisant à une consultation gynécologique au cours de laquelle une masse bourgeonnante et saignante du col utérin a été mise en évidence. Une première biopsie réalisée le 9 janvier 2024 a suggéré un processus tumoral utérin, conduisant à une relecture anatomopathologique et à de nouvelles biopsies le 25 janvier 2024. Le 30 janvier 2024, l'examen histopathologique a évoqué un carcinome à cellules claires du col de l'utérus, nécessitant une confirmation immunohistochimique. Le 19 février 2024, la tomodensitométrie thoraco-abdomino-pelvienne n'a pas révélé de métastase. Le 22 février 2024, l'étude immunohistochimique a confirmé le diagnostic. Le 27 février 2024, l'imagerie par résonance magnétique (IRM) abdomino-pelvienne a montré une tumeur cervicale localement avancée de stade IIIA mesurant 36 x 50 mm avec des adénopathies iliaques externes gauches. Entre le 28 avril et le 4 juin 2024, la patiente a reçu une radiochimiothérapie concomitante. Le 16 juin 2024, l'IRM de contrôle a montré une disparition quasi complète de la tumeur. Le 18 juillet 2024, une colpohystérectomie élargie a été réalisée, et le 1^er^ août 2024, l'examen anatomopathologique de la pièce opératoire n'a retrouvé aucun reliquat tumoral.

**Résultats cliniques:** à l'examen physique, la patiente présentait une pâleur, avec des signes vitaux normaux. L'examen gynécologique a révélé une masse bourgeonnante du col de l'utérus, saignant au contact, justifiant la réalisation de biopsies cervicales.

**Figure 1:**
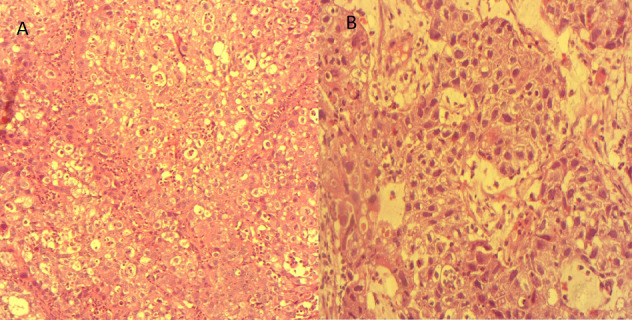
aspect histologique tumoral à faible et moyen grossissement; A) architecture solide de la tumeur, composée de cellules éosinophiles et claires, x100; B) architecture tubulaire de la tumeur, constituée de cellules éosinophiles et claires, x200

**Figure 2:**
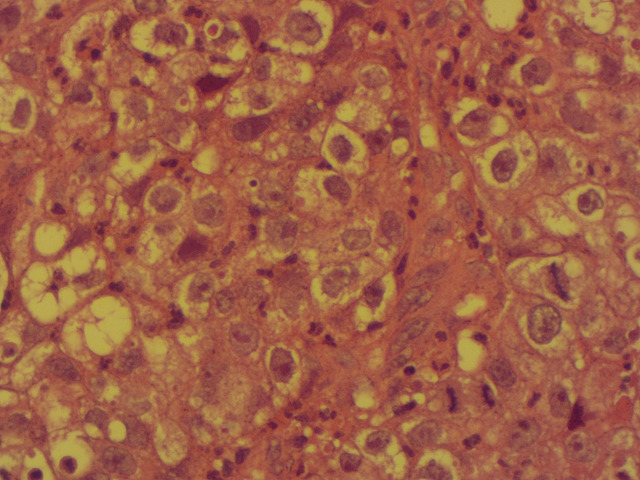
détails cytologiques de la prolifération tumorale; cellules éosinophiles et claires hautement atypiques, avec des noyaux hyperchromatiques et de nombreuses mitoses, x400

**Figure 3:**
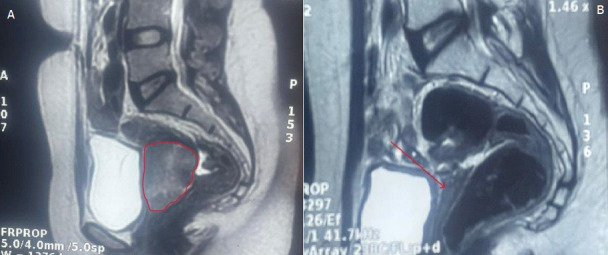
imagerie par résonance magnétique pelvienne en coupe sagittale avant et après traitement; A) IRM avant traitement montrant une tumeur du col de l'utérus localement avancée, stade IIIA, mesurant 36 mm x 50 mm (cercle rouge); B) IRM après radiochimiothérapie montrant une disparition quasi-totale de la tumeur (flèche rouge)

**Figure 4:**
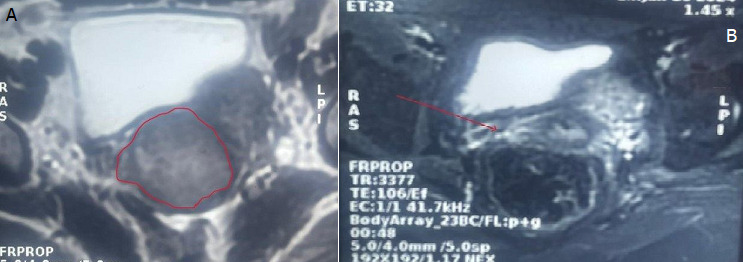
imagerie par résonance magnétique pelvienne en coupe axiale avant et après traitement; A) IRM avant traitement montrant une tumeur du col de l'utérus localement avancée, stade IIIA, mesurant 36 mm x 50 mm (cercle rouge); B) IRM après radiochimiothérapie montrant une disparition quasi-totale de la tumeur (flèche rouge)

**Démarche diagnostique:** la démarche diagnostique a débuté par un examen gynécologique complet suivi de biopsies cervicales initiales. En raison d'une discordance entre les données cliniques et la première biopsie, une relecture anatomopathologique associée à de nouvelles biopsies a été demandée. L'examen histologique a montré une prolifération tumorale faite de massifs solides avec de rares structures glandulaires, constituée de cellules polygonales à cytoplasme clair ou éosinophile modérément abondant, avec des atypies cytonucléaires marquées et de nombreuses mitoses. Le stroma était fibro-œdémateux avec congestion vasculaire et suffusions hémorragiques, sans signe de différenciation malpighienne (Figure 1, Figure 2). L'étude immunohistochimique a montré une expression nucléaire focale de p16, sans expression de p63 ni de P40, confirmant un adénocarcinome à cellules claires du col de l'utérus. La tomodensitométrie thoraco-abdomino-pelvienne n'a pas mis en évidence de métastases, tandis que l'IRM abdomino-pelvienne a montré une tumeur cervicale localement avancée avec adénopathies iliaques externes gauches (Figure 3, Figure 4). Les diagnostics différentiels envisagés comprenaient un carcinome épidermoïde du col de l'utérus et d'autres types d'adénocarcinomes cervicaux. Les données histopathologiques et immunohistochimiques ont permis d'écarter ces hypothèses et de retenir le diagnostic final de carcinome à cellules claires du col de l'utérus.

**Facteurs pronostiques:** la taille de la tumeur (36 mm x 50 mm), le stade avancé localement (stade IIIA), et l'absence de métastases détectées par TAP sont des facteurs importants dans l'évaluation du pronostic. La présence d'adénopathies iliaques externes gauches constitue également un facteur pronostique défavorable.

**Figure 5:**
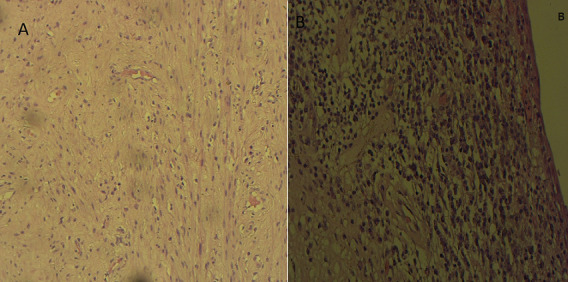
étude histologique de la pièce opératoire après traitement: A) absence de reliquat tumoral identifiable, associée à une fibrose marquée, x100; B) absence de reliquat tumoral décelable, avec infiltrat inflammatoire moyennement abondant et diffus, x200

**Intervention thérapeutique:** la prise en charge thérapeutique a reposé sur une radiothérapie externe délivrée à une dose totale de 70 Gy en 35 séances, associée à une chimiothérapie hebdomadaire à base de cisplatine (40 mg/m^2^), administrée entre le 28 avril et le 4 juin 2024. La curiethérapie n'a pas pu être réalisée en raison d'un défaut technique, limitant l'approche thérapeutique classique. Malgré cette contrainte, une réponse tumorale quasi complète a été obtenue. Une colpohystérectomie élargie a été réalisée le 18 juillet 2024, sans complication peropératoire ou postopératoire, et l'examen anatomopathologique de la pièce opératoire n'a montré aucun reliquat tumoral (Figure 5).

**Suivi et résultats des interventions thérapeutiques:** la patiente a bien toléré l'ensemble des traitements, sans effet indésirable significatif rapporté. Le suivi clinique et radiologique n'a pas mis en évidence de récidive. L'hypothyroïdie était bien équilibrée sous traitement substitutif.

**Perspectives de la patiente:** la patiente a exprimé son point de vue concernant sa prise en charge: «J'attendais plutôt un enfant, mais j'ai fait une fausse couche aboutissant à la détection de cette tumeur. Tout ce que fait Dieu est bon, Dieu merci. Je suis très reconnaissante pour le traitement opportun que j'ai reçu».

**Consentement éclairé:** le consentement éclairé a été obtenu auprès de la patiente pour la publication de ce rapport de cas ainsi que de toutes les images qui l'accompagnent, et peut être fourni sur demande.

## Discussion

Le CCC est un cancer rare, représentant environ 4% des adénocarcinomes cervicaux. Historiquement, il est associé à l'exposition intra-utérine au diéthylstilbestrol (DES) dans environ deux tiers des cas. Néanmoins, des cas ont été signalés chez des femmes adultes et des enfants sans antécédents d'exposition au DES, comme illustré dans notre observation [1]. L'étude de Herbst *et al*. a initialement établi le lien entre l'exposition au DES pendant la grossesse et l'apparition de ce type de cancer, principalement chez des femmes jeunes exposées in utero [4]. La survenue de CCC chez des patientes sans exposition connue au DES suggère l'intervention d'autres facteurs étiologiques potentiels, décrits dans la littérature (surexpression de p53, HPV, bcl-2, instabilité des microsatellites) [1]. Une analyse comparative a montré que l'âge moyen des patientes sans exposition au DES était de 47 ans [5], tandis qu'une autre étude a rapporté un âge médian de 38 ans [6], plaçant notre patiente de 40 ans dans la tranche d'âge habituellement décrite.

Les manifestations cliniques du CCC sont souvent non spécifiques, dominées par des saignements vaginaux irréguliers, comme observé chez notre patiente après un avortement spontané [1,7]. Cette présentation peu spécifique peut retarder le diagnostic, les symptômes étant parfois attribués à des troubles gynécologiques bénins. Les carcinomes à cellules claires sont ainsi souvent diagnostiqués à un stade légèrement plus avancé que les autres adénocarcinomes cervicaux [8]. Dans notre cas, la discordance initiale entre les données cliniques et la première biopsie a constitué une difficulté diagnostique, nécessitant une relecture anatomopathologique et de nouvelles biopsies, ce qui souligne l'importance de la collaboration clinico-pathologique dans la prise en charge de tumeurs rares [6,9]. Le point fort principal de ce rapport réside dans la confirmation diagnostique reposant sur l'histologie, l'immunohistochimie et l'imagerie (TAP et IRM), permettant une stadification et une stratégie thérapeutique adaptées.

Le diagnostic anatomopathologique repose sur des caractéristiques morphologiques spécifiques, et l'immunohistochimie joue un rôle déterminant pour écarter les diagnostics différentiels, notamment le carcinome épidermoïde du col utérin. L'absence d'expression de p40 et p63, associée à une expression focale de p16, a permis de confirmer le diagnostic de CCC dans notre cas [10]. Concernant l'hypothyroïdie, aucune relation causale ne peut être conclue dans le cadre d'un rapport de cas; les données disponibles concernent surtout d'autres cancers, avec un risque potentiellement accru en cas d'hypothyroïdie subclinique non traitée et un possible effet protecteur du traitement substitutif [7]. Ainsi, l'évocation de cette comorbidité doit être interprétée avec prudence et ne constitue pas une conclusion étiologique.

En raison de la rareté du CCC, les stratégies thérapeutiques sont généralement extrapolées de celles des carcinomes épidermoïdes et des autres adénocarcinomes cervicaux [8]. Le traitement standard des cancers du col de l'utérus localement avancés repose sur la radiochimiothérapie concomitante suivie d'une curiethérapie utérovaginale [1-4]. Dans notre cas, l'absence de curiethérapie pour des raisons techniques constitue une limite majeure, avec un impact potentiel sur le contrôle local et la préservation de la fertilité [1,5]. Malgré cela, la radiochimiothérapie suivie d'une colpohystérectomie élargie a permis une réponse complète, sans reliquat tumoral. Le pronostic du CCC dépend du stade, de la taille tumorale et des caractéristiques histologiques, avec un taux de survie à cinq ans estimé à environ 40% [9]. Les limites de ce rapport tiennent au caractère isolé du cas et à la durée de suivi, qui restreignent la portée des conclusions. Néanmoins, ce cas justifie de conclure à la faisabilité d'une prise en charge multidisciplinaire efficace d'un CCC localement avancé dans un contexte à ressources limitées, sous réserve d'un suivi prolongé.

## Conclusion

Ce cas d'adénocarcinome à cellules claires du col de l'utérus chez une femme de 40 ans sans exposition au DES illustre la complexité diagnostique de cette entité rare et l'importance d'une collaboration multidisciplinaire. Il montre qu'une prise en charge combinant radiochimiothérapie et chirurgie peut permettre une réponse favorable, même en l'absence de curiethérapie pour des raisons techniques. Ce rapport de cas souligne la faisabilité d'une prise en charge efficace des cancers cervicaux rares dans des contextes à ressources limitées et contribue à enrichir les données disponibles dans la littérature africaine.
